# Genetic variants of the transporter SLC22A4 affect the abundance and survival of *Fusobacterium nucleatum* in colorectal cancer

**DOI:** 10.1080/19490976.2026.2681818

**Published:** 2026-06-05

**Authors:** Samah Chouaibi, Veronica Fertitta, Silvia Porreca, Leila Njim, Antonella Carcagnì, Gabriele Toietta, Gianluca Canettieri, Khadija Zouari, Maha Mastouri, Yosr Kadri, Giovambattista Pani

**Affiliations:** a Department of Translational Medicine and Surgery, Faculty of Medicine, Università Cattolica del Sacro Cuore, Rome, Italy; b Laboratory of Transmissible Diseases and Biologically Active Substances LR99ES27, Faculty of Pharmacy, University of Monastir, Monastir, Tunisia; c Department of Pathological Anatomy and Cytology, Fattouma Bourguiba University Hospital, Monastir, Tunisia; d Facility of Epidemiology and Biostatistics–Gemelli Generator, Fondazione Policlinico Universitario A. Gemelli IRCCS, Rome, Italy; e Tumor Immunology and Immunotherapy Unit, IRCCS Regina Elena National Cancer Institute, Rome, Italy; f Department of Molecular Medicine, Sapienza University of Rome, Rome, Italy; g Colorectal Diseases Research Unit, Faculty of Medicine, University of Monastir, Monastir, Tunisia; h Fondazione Policlinico Universitario A. Gemelli IRCCS, Rome, Italy

**Keywords:** Colorectal cancer, *Fusobacterium nucleatum*, microbiota, OCTN1, genetic variants, cancer stem cells, innate immunity

## Abstract

The intestinal microbiota influences colorectal cancer (CRC) development, but its interactions with the host's genetic profile during tumorigenesis are poorly understood. We quantified the CRC-associated pathobiont *Fusobacterium nucleatum* (*F. nucleatum*) and the commensal *Escherichia coli* (*E. coli*) in 99 cases of archival colorectal cancer and adjacent normal mucosa. Tissues were genotyped for the 503F variant of the Organic Cation Transporter OCTN1/SLC22A4. Colorectal cancer stem cells engineered to express the variant were infected with *F. nucleatum* in vitro*. F. nucleatum* was similarly present in colorectal cancer tissues and the adjacent normal mucosa, but the *F. nucleatum*/*E. coli* ratio was significantly higher in tumors (303.82 vs 30.86, *p*-value = 0.0396), in a fashion that steadily increased with the number of mutant SLC22A4 alleles (23.48, 159.56, and 211.03 for 0, 1, or 2 T alleles; *p* = 0.0215). Colon cancer spheroids overexpressing the 503F variant, but not the wild-type allele, displayed attenuated inflammatory response to *F. nucleatum* and impaired bacterial clearance, mechanistically linking SLC22A4 function and intratumoral *F. nucleatum* abundance. Thus, genetic variants of the intestinal carrier SLC22A4 shape the intratumor microbiota in favor of a pro-carcinogenic pathobiont by dampening innate immunity and increasing the tolerance of cancerous cells to bacterial invasion.

## Graphical abstract

**Figure:**
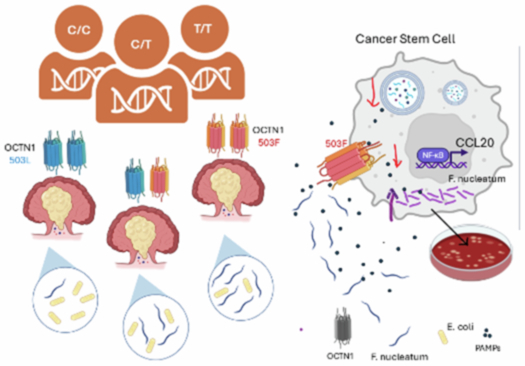

## Introduction

Colorectal cancer (CRC) is the second leading cause of cancer-related deaths worldwide and ranks third in terms of incidence.^1^ CRC develops from epithelial cells in the large intestine due to the accumulation of genetic and epigenetic disturbances^2^ ^,^ ^3^ diverse risk factors, including the environment, lifestyle, inflammation, personal and familial history of inflammatory intestinal diseases and benign adenomatous polyps, all contribute to the evolution of this heterogeneous disease.^4^ Recently, the development of CRC has been increasingly linked to a modified composition of the intestinal microbiota, marked by a decrease in beneficial species of the *Firmicutes* phylum and an increase in pro-inflammatory or pathogenic species belonging to *Proteobacteria* and *Fusobacteria*.^5^

*F* *usobacterium* *nucleatum*, a non-sporulated, anaerobic Gram-negative bacterium,^6^ inhabits the buccal cavity in the healthy population as a mutualist,^7^ but it has also been shown to play a role in a variety of disorders, including gastrointestinal illnesses.^8^ Most importantly, numerous studies have reported the presence of *F. nucleatum* within the tumor tissue of patients with colorectal cancer, and a higher abundance of *F. nucleatum* in cancerous compared to healthy tissues. Moreover, higher levels of *F. nucleatum* in CRC are linked to a lower survival rate.^9-12^ Mechanistically, *F. nucleatum*'s ability to enter the bloodstream, colonize the intestinal microbiota, and infect epithelial cells through its virulence factors (like the adhesins FadA and Fap2) explains its enrichment in colorectal cancer.^13^ ^,^ ^14^ By additional complex mechanisms, only in part elucidated, *F. nucleatum* aids in the proliferation and spread of colorectal cancer cells while suppressing the T cell-dependent immune response,^15^ and promotes inflammation through TLR4 receptor engagement,^16^ activation of the master proinflammatory transcription factor NF-κB, and the induction of an array of cytokines and humoral factors overall conducive to local tumor growth and metastasis.^17^ It is also increasingly clear that at least some of these pathogenic effects involve the cooperation/antagonism of *F. nucleatum* with other bacterial species, and more generally, *F. nucleatum*-related perturbations in the local bacterial communities, in accordance with the “alpha-bug” model.^18^ Thus, the multifaceted interactions between *F. nucleatum* and other microorganisms add a further layer of complexity to interpreting the potential role of this pathobiont and its associated microbiota as truly causative or, instead, bystanders in colorectal carcinogenesis.^19^

While it is well established that an individual's genetic profile can predispose them to the development and progression of CRC, less is known about how microbiota changes contribute to genetic susceptibility to the disease.^20^ This information is crucial to factor in microbes, particularly *F. nucleatum*, in the individual vulnerability to cancer.

The organic cation transporter SLC22A4 is well known for its potential role in genetic susceptibility to inflammatory bowel disease. Moreover, we have identified the 503F variant of SLC22A4 as a predisposing genetic factor for CRC, particularly in young individuals and in colitis-associated cancer.^21^ The mechanistic underpinnings of these yet solid genetic associations remain largely elusive: while the established role as the intestinal transporter of the dietary antioxidant Ergothioneine (ERGO) may certainly link an altered SLC22A4 activity to increased oxidative stress, enhanced inflammation and possibly tumor promotion, a possible wider role for this transporter in immunological regulation is also possible, based on recent evidence that several solute carrier proteins closely related to SLC22A4 are directly involved in innate immune responses, specifically through their crosstalk with pattern recognition receptors like NOD-like and Toll-like receptors.^22^ Accordingly, SLC22A4 has been found to inhibit IL-1β expression in glial cells, and more relevant to the present work, the wild-type carrier and even more its L503F variant potentiate IL-1β release by human macrophages in response to bacterial Peptidoglycan (P. Puca and G. Pani, Inflamm. Bow Dis., *in press*). Thus, it is conceivable that SLC22A4 participates in communication between the intestinal microbiota and mucosal cells (both epithelial and inflammatory) by transporting bacteria-derived antigens and metabolites that can influence epithelial cell proliferation as well as local inflammatory and immune responses. Reciprocally, this carrier may affect the intestinal microbiota's composition, influencing the contact with its host and regulating the epithelium's immune response.^23^

In the present work, we set out to investigate the connection linking microbiota, genetic susceptibility, and innate immunity in colorectal carcinogenesis. More specifically, we focused on *F. nuclesatum* as the prototype of a CRC-associated pathogen, and SLC22A4 as a host factor genetically linked to colorectal cancer and mechanistically involved in microbe-host communication.

## Materials and methods

### Patients

Ninety-nine patients (49 men and 50 women, aged 28 to 92) with histologically verified colon malignancies were included in the study. The Formalin-fixed, paraffin-embedded (FFPE) tumor tissues and adjacent colon normal mucosa were obtained from the Anatomy and Cytology Pathology service at Fattouma Bourguiba University Hospital in Monastir, Tunisia, between January 2022 and July 2023. Patients with colon tumors other than adenocarcinoma, concurrent cancers from other organs, and those who had received chemotherapy or radiation therapy prior to surgery were excluded. Clinical and pathological information (age, sex, tumor location, macroscopic tumor type, pT/pN categories, and tumor grade) was obtained from the hospital's data bank. The study's protocol was approved by the institutional ethical committee (University of Monastir, IORG 0009738 N 101/OMB 0990-0279).

### Real-time PCR

For detecting and quantifying *F. nucleatum* and *E. Coli* in tumor and normal mucosa samples, DNA was extracted from FFPE tissues (tumors and adjacent normal colonic mucosa) by a standard procedure and quantified by a microvolume spectrophotometer (NanoDrop, Thermo Fisher Scientific, Waltham, MA, US). *F. nucleatum* and *E. coli* DNA abundance was assessed by quantitative real-time amplification (qPCR) using the SYBR Green Master Mix kit (Applied Biosystems, Whaltam, MA, US) in a CFX96 qPCR thermocycler (Bio-Rad, Hercules, CA, US) and the following primer sets: *F. nucleatum* genus forward primer, 5ʹ-AAGCGCGTCTAGGTGGTTATGT-3ʹ; *F. nucleatum* genus reverse primer, 5ʹ-TGTAGTTCCGCTTACCTCTCCAG-3ʹ (sequence ID: CP053468.1); *E. coli* genus forward primer: 5ʹ-CAACGAACTGAACTGGC AGA-3ʹ; *E. coli* genus reverse primer: 5ʹ-CATTACGCTGCGATGGAT- 3ʹ (Sequence ID: OZ039253.1).

To detect bacterial DNA, a 20 μL reaction mixture containing 100 ng of DNA, 10 µM of primers, and 10 μL of SYBR Green Master Mix (Applied Biosystems) was prepared. The amplification process consisted of an initial 10-minute phase at 95 °C, and 40 cycles, each including 15 seconds denaturation at 95 °C, and 60 seconds hybridisation–elongation at 60 °C. Purified microbial DNA was used to confirm the linear correlation between cycle threshold (Ct) values and the amount of input DNA (plotted on a log2× scale).

The Ct values for *F. nucleatum* and *E. coli* were normalized to the amount of human genomic DNA in each reaction using the human Prostaglandine Transporter (SLCO2A1) reference gene (SLCO2A1 gene forward primer: 5ʹ-ATCCCCAAAGCACCTGGTTT-3ʹ; SLCO2A1 gene reverse primer: 3ʹ-AGAGGCCAAGATAGTCCTGGTAA-5ʹ) and the 2^−^^ΔCt^ calculation method.

To assess Slc22a4 mRNA expression in CSC cells, total mRNA (QIAzol Lysis Reagent, QIAGEN, Hilden, Germany) was copied into cDNA (SensiFAST™ cDNA Synthesis Kit, Meridian Biosciences, Cincinnati, OH, US) and amplified with the following primer pair:

Slc22a4 F 5ʹ-TGGACCTGTTCAGGACTCGGAA; Slc22a4 R 5ʹ-TAGGAGCATCCAGAGACAGAGC.

The PCR products were electrophoresed on a 2% Agarose gel containing 1:10000 GelRed® Nucleic Acid Gel Stain (Biotium, Fremont, CA, US) and visualized under UV light. A no-Reverse Transcriptase control was included to rule out amplification of contaminant genomic DNA.

### Patient genotyping for the rs1050152 (Slc22a4 c.1672T) SNP

Genomic DNA obtained from normal colonic tissues was analysed using a predesigned probe set (TaqMan Assay ID, C___3170459_30) and the TaqMan Genotyping Master Mix kit, both from Applied Biosystems. Each real-time PCR reaction was prepared in a 25 µL volume, which included 12.5 µL of TaqMan master mix, 1.25 µL of the genotyping oligo mix, 20 ng of genomic DNA, and nuclease-free water to reach the final volume. The reaction included a 10-minute initial denaturation step at 95 °C, and 40-cycles of amplification (15 seconds at 95 °C, and 1 minute at 60 °C each). Allele detection was carried out according to the manufacturer's instructions, using the CFX96 qPCR device (Bio-Rad, Hercules, California, United States). Patients were classified based on the number of major (1672c) and minor (1672t) alleles as C/C, C/T, and T/T (homozygous variant).

### In vitro studies

#### Cell lines

The primary spheroid colon cancer culture CSC-P was initially derived at Istituto Superiore di Sanità, Rome, Italy, and made available to GBP under a Material Transfer Agreement. The procedure of isolation and characterization is described in detail in De Angelis et al.^24^ Briefly, surgical specimens of primary CRCs were digested in Collagenase II + DNAse for 1 hour at 37 °C and resuspended in a serum-free defined growth medium containing 10 ng/ml human bFGF, 20 ng/ml human EGF. Spheroids forming in 2–4 weeks from cell seeding were expanded in CSC medium [Advanced DMEM/F12, supplemented with Vitamin A-free B27 (Life Technologies, Carlsbad, CA, US), 10 mM Nicotinamide (Merck, Darmstadt, Germany), 1 μM RhoK inhibitor Y-27632 (Tocris Biosciences, Bristol, UK; cat. #1254), 6 g/L glucose, 2 μg/ml Heparin (StemCell Technology, Vancouver, BC, Canada; cat.#07980), 10 ng/ml bFGF and 20 ng/ml hEGF (StemCell Technologies)], and passaged weekly. Spheroid aggregates were dissociated by gentle trypsinization; cell suspensions were passed through a 70 μm-pore size strainer (FlowMi^TM^, SP Bel-Art, Wayne, NJ, U.S.A.; cat.#136800070) and re-seeded at 1.5 × 10^5^ cells/ml in 25 cm^2^ ultra-low attachment tissue culture flasks.

293 T cells (#CRL-3216) were obtained from ATCC and routinely maintained in Dulbecco's Modified Eagle's Medium (DMEM) containing 10% heat-inactivated Fetal Bovine Serum (FBS).

#### Bacteria

*F. nucleatum* subsp. *nucleatum* Knorr 25586™ was obtained from American Type Culture Collection, ATCC, Manassas, VA, US. Bacteria were routinely grown in Tryptic Soy Broth—Peptone (TSBP) supplemented with 0.25% v/v Cysteine.^25^ Liquid cultures were titrated by Colony Forming Unit (CFU) counting on sheep blood agar plates incubated in anaerobic pouches (GasPak™ EZ, Becton, Dickinson, Franklin Lakes, NJ, US).

#### Plasmids

The plasmids encoding the human SLC22A4 variants 503 L and 503F (tagged with a myc epitope at the C-terminus) in the pCDNA3.1 vector backbone, and the full-length human NOD2/CARD15 cDNA C-fused with eGFP were a generous gift of Prof. Katherine Siminovitch (Mount Sinai Hospital, Toronto). The lentiviral construct encoding the mouse Slc22a4 cDNA (NM_019687) tagged at the C terminus with monomeric (m)GFP (#SKU MR208779L4) was purchased from OriGene (Rockville, MD, US); the MISSION® pLKO.1-puro empty control vector (cat. #SHC016) was obtained from Sigma/Merck. The lentiviral transfer vectors for SLC22A4-myc 503 L and 503F SLC22A4 were obtained by directionally cloning the BamHI-XhoI inserts excised from the original pCDNA3.1-based plasmids onto the BamHI-SalI sites of the pCCL.sin.cPPT third-generation, HIV-1-based self-inactivating (SIN) lentiviral vector backbone. Lentiviral supernatants were produced according to Tiscornia et al.^26^, with minor changes; the plasmid mixture containing the transfer vector, pMDL, pRev, and pVSVG, was introduced into HEK-293T cells by Calcium Phosphate precipitation. Supernatants from the second and third day post-transfection were pooled and concentrated 100 times by ultracentrifugation (72000 g for 2 hours at 20 °C). Pooled supernatants from a 1–2 × 10 cm Petri dish (4 × 10^6^ packaging cells) were used to infect 2 × 10^5^ CSC cells in 2 ml complete CSC medium. To increase the infection efficiency, cells were co-centrifuged with lentiviral particles at RT for 2.5 h at 2500 g (“spinoculation”), in the presence of 8 μg/ml Polybrene (Merck/Sigma-Aldrich).

#### Cell transfection

Transfection of 293-T cells was carried out in 6-well plates using the non-liposomal lipid reagent EFFECTENE (QIAGEN, Hilden, Germany) according to the manufacturer's recommendations. Briefly, 10^6^ cells/well were transfected with 1 μg plasmid DNA, complexed with 4 μL enhancer and 10 μL lipid reagent, in complete medium. Cells were collected 72 hours later for further processing.

#### Antibodies

anti c-myc (clone 9E10, cat. # sc-40) and anti GFP (clone B2, cat. # sc-9996) mouse monoclonal antibodies were from Santa Cruz Biotechnology (Dallas, TX, US). Anti LC3B (clone D11, cat. # 3868) and anti-beta-actin (clone 8H10D10, cat. # 3700) were from Cell Signaling Technologies (Danvers, MA, US). The anti-*F. nucleatum* polyclonal rabbit antiserum (cat. # ANT0084) was purchased from Diatheva (Cartoceto, PU, Italy). The HRP-conjugated goat anti-mouse IgG (H + L) and goat anti-rabbit IgG (H + L) antisera were obtained from Bio-Rad (cat. #1706516 and #1706515, respectively).

#### Cell Infection in vitro and Gentamicin protection assay

Spheroids were dissociated into single cells, counted, and reseeded at 2-2.5 × 10^5^/well/500 µL complete CSC medium without antibiotics in ultra-low attachment 24-well clusters. *F. nucleatum* was revived from titrated glycerol stocks at 37 °C for 30 minutes, washed in PBS, and resuspended in CSC medium at 10^9^ CFU/ml. 40–50 µL of bacterial suspension (MOI 200), or just CSC medium, were added to the wells. After 6 hours, Gentamicin was added at 100 µg/ml, and cultures were incubated for a further 18 hours.

Following 24-hour incubation with bacteria (6 hours + 18 hours with Gentamicin), cells were spun down, washed once in PBS, and resuspended in 80 µL of PBS containing 0.01% TRITON X-100 to release intracellular bacteria. Forty microliters of lysate were immediately plated onto blood agar plates and incubated in anaerobic pouches at 37 °C for 3–5 d until visible colonies appeared. The remaining 40 µL were mixed with 8 µL of 6× Laemmli buffer, boiled, and subjected to western blotting.

#### Cell lysis and immunoprecipitation

For co-immunoprecipitation studies, cell pellets (10^6^ cells) were lysed in 1 ml of cold lysis buffer (50 mM Tris–HCl pH8.0; 150 mM NaCl; 5  mM EDTA; 0.05% Na+ Azide) containing 1% (v/v) Triton-X100, protease, and phosphatase inhibitors. After 15 minutes of incubation on ice, tubes were spun down (14,000 rpm for 10 minutes at 4 °C) to remove cell debris and unlysed nuclei, and supernatants were precleared with empty Protein A/G se-pharose beads (100 μL of a 10% v/v slurry) for 1 h at 4 °C on a rocking plate. After centrifugation, 1/20 of the supernatant was kept for western blot analysis (input), and 19/20 were incubated with 1 μg of antibody myc and 100 μL of protein A/G slurry for 16 hours in rotation at 4 °C. Sepharose-bound immunocomplexes were collected by brief centrifugation (14,000 rpm for 30 seconds), washed 4–5 times in lysis buffer with inhibitors, eluted in Laemmli buffer and analyzed by western blotting.

#### Western blotting

Protein samples in Laemmli buffer were heated at 95 °C for 5 minutes, applied to SDS–PAGE, and electroblotted onto a nitrocellulose membrane. Membranes were blocked 5% w/v skim milk in TBS-T (1 hour at RT) and incubated with primary reagents (1:1000 in TBST + 3% w/v skim milk) O/N at 4 °C on a rocking plate. Immunocomplexes were visualized by incubating the filters for 1 hour at RT with Goat Anti-Mouse IgG (H + L)-HRP or Goat Anti-Rabbit IgG (H + L)-HRP (1:5000 in TBST), followed by enhanced chemiluminescence (Westernbright™ ECL, Advansta, San Jose, CA, US: cat. # K-12045). Filters were imaged in an Alliance Q9® advanced chemiluminescence station (Uvitec, Cambridge, UK).

#### Statistical analysis

The Shapiro-Wilk test was applied to verify the normal distribution of data. Because of the skewed quantitative variables distribution, the Wilcoxon signed-rank test, the Mann–Whitney test, and the Kruskal–Wallis test were used for single (paired or unpaired) and multiple comparisons, respectively. A Kruskal–Wallis test followed by a post hoc Mann–Whitney U test was performed to compare data from different groups. Bonferroni correction was applied to adjust for the increased risk of Type I errors in multiple comparisons (typically three pairwise comparisons for three genotypes). Categorical variables were analyzed using the chi-square test and/or Fisher's exact test, as appropriate.

Multivariable regression models were used to assess the effect of SLC22A4 genotype relative to other clinico-pathological variables on the outcome (*F. nucleatum/E. coli* “double” ratio). Statistical analyses were performed using free computational websites (VassarStats.net; or https://epitools.ausvet.com.au/) and the statistical software R (R, CRAN, 2025). A *p*-value of less than 0.05 (two-tailed) was considered statistically significant.

## Results

### Increased *F. nucleatum*-to-*E.* *coli* ratio in CRC tissue compared to the adjacent healthy mucosa

**Figure 1.**
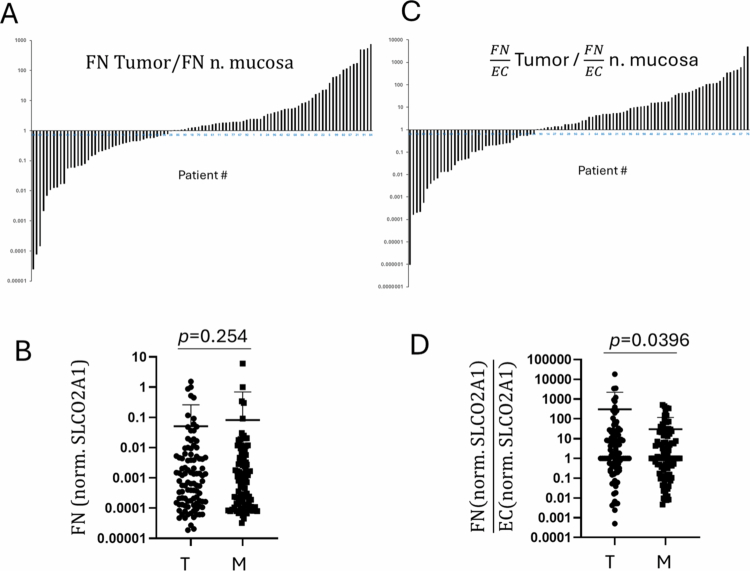
qPCR analysis of *F. nucleatum* and *E. coli* abundance in colorectal cancers and matched normal tissues (*n* = 99). (A) and (C): column histograms depicting the tumor-to-normal mucosa ratios for *F. nucleatum* (FN, panel A) or *F. nucleatum/E. coli* ( $\frac{\mathrm{FN}}{\mathrm{EC}})\;(\mathrm{panel}\;C)\;\mathrm{in}\;\mathrm{each}$ patient. Values were ranked in ascending order from left to right. (B) and (D): Dot plots illustrating the distribution of *F. nucleatum* abundance (panel B) and the *F. nucleatum/E. coli* ratio (panel D) in tumors (T) and matched normal tissues (M). Bacterial abundances were normalized to host DNA using hSLCO2A1 as a reference. The *F. nucleatum*/*E. coli* ratio was significantly higher in tumor tissues compared to normal mucosa (303.82 vs 30.86; *p* = 0.0396, Wilcoxon signed-rank test). Conversely, straight *F. nucleatum* was overall lower and showed no significant difference.

We used quantitative PCR (qPCR) to assess the abundance of *F. nucleatum* and *E. coli* in Formalin-fixed, paraffin-embedded colorectal tumor biopsies and adjoining normal mucosa. The total amount of extracted DNA was measured, and bacterial loads were normalized to host DNA using the human Prostaglandin Transporter (PGT/SLCO2A1) gene as a reference. DNA amplification results confirmed that the majority of tumor tissues and the surrounding mucosa harbored *F. nucleatum* and *E. coli*, with significant interindividual variation in their abundance. Unlike reported in several previous studies, there was no significant difference in the abundance of *F.nucleatum* between the cancer tissue and the adjacent normal mucosa (0.050 vs 0.082, *p* = 0.254 by two-tailed Wilcoxon signed rank test), arguing against a tumor-specific absolute enrichment of this bacterial species (Figure 1, panels A and B). However, compared with normal tissue, the *F. nucleatum-to-E. coli* ratio was significantly higher in tumors (303.82 vs 30.86; *p* = 0.0396, two-tailed Wilcoxon signed-rank test), consistent with a specific microbiological imbalance in the tumoral microenvironment (Figure 1C and D).

### SLC22A4 variants impact the CRC microbiota

**Figure 2.**
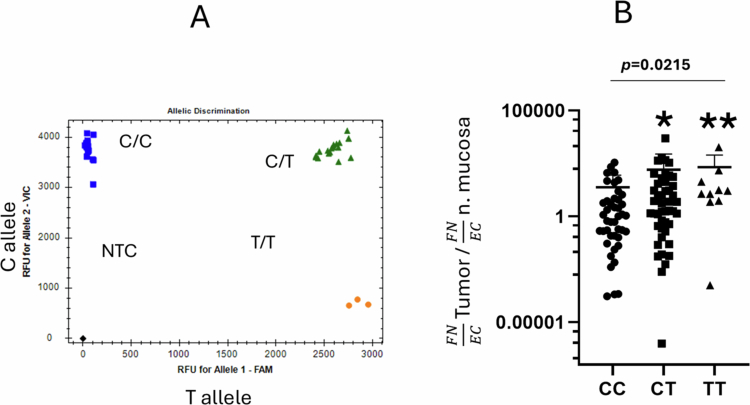
Association Between Tumor Tissue Bacterial Abundance and SLC22A4 Genotype: (A). Allele discrimination plot representative of one of several rs1050152 genotyping sessions. NTC denotes no template control. (B). Dot-plot illustrating the distribution of the FN/EC tumor-to-normal mucosa ratios across the three SLC22A4 genotypes. The “double ratio” of *F. nucleatum*/*E. coli* increased progressively with the number of mutant SLC22A4 alleles (23.48, 159.56, and 211.03 for 0, 1, or 2 T alleles, respectively; *p* = 0.0215, Kruskal–Wallis test). Pairwise comparisons of C/T versus C/C (*p < 0.05) and T/T versus C/C (**p < 0.001) were also significant by the Mann–Whitney test.

Having established that a higher *F. nucleatum*-to-*E. coli* ratio is characteristic of cancer tissue compared to normal mucosa in our CRC cohort, we asked whether SLC22A4 variants may further shape this difference. Patients were genotyped for rs5015152 (*Slc22a4* c1672t) by qPCR (TaqMan, Assay ID: C-3170459-30) and divided into three groups (C/C, *n* = 43; C/T, *n* = 46; T/T, *n* = 10) accordingly (Figure 2A). No significant correlations were found between SLC22A4 genotype and patient demographics or disease location and stage/severity, although TT individuals tended to be older and distant metastases appeared more frequently in variant-bearing (CT + TT) individuals (Table 1).

**Table 1.** Patient demographics and clinical characteristics per genotype.

		Total (*n* = 99)	CC (*n* = 43)	CT (*n* = 46)	TT (*n* = 10)	*p*
Sex	F	50	25	19	6	*p* = 0.240 (Fisher Exact probability test 2 × 3)
	M	49	18	27	4	
Age	Mean	64.74	65.81	62.37	71.00	*p* = 0.114 ANOVA
	SD	12.71	12.42	12.84	11.67	
Tumor location	Right/transverse	33	16	11	6	*p*= 0.633 (Fisher Exact Probability test 2 × 4, CC vs CT + TT)
	Left	46	17	26	3	
	Rectum	18	9	8	1	
	Not specified	2	1	1	0	
Advanced disease (stages III and IV)	Yes	46	18	24	4	*p* = 0.42 (Chi-square CC vs CT + TT 2X2)
	No	53	25	22	6	
Distant metastases	Yes	18	5	11	2	*p* = 0.138 (Chi-square CC vs CT + TT 2 × 2)
	No	81	38	35	8	

Abbreviations: *n*: number of participants, F: female, M: male, SD: standard deviation, *p* < 0.05 indicates statistical significance.

Analysis of bacterial abundance by genotype in tumor tissues revealed a tendency for patients carrying one or two mutant alleles (CT + TT) to have lower *E. coli* and a higher *F. nucleatum/E. coli* ratio compared to the C/C group (*p* = 0.161 and *p* = 0.274, respectively, Mann-Whitney directional test; supplementary Figure 1). Interestingly, when the three groups were compared for the double ratio (*F. nucleatum*/*E. coli* in tumor vs healthy mucosa) a clear microbe/genotype interaction emerged, with the mean value growing steadily with the number of mutant SLC22A4 alleles (23.48, 159.56, and 211.03 for 0, 1, or 2 alleles T, respectively; *p* = 0,0215, Kruskal–Wallis test) (Figure 2B). A multivariable regression model confirmed that genotype was the only variable significantly associated with the outcome (double ratio). Individuals carrying the T/T genotype showed a strong and statistically significant increase in the double ratio compared with the reference genotype (*β* = 11.10, SE = 3.16, *t* = 3.51, *p* = 4.5 × 10⁻⁴, 95% CI: 4.90–17.30). The C/T genotype was associated with a positive effect (*β* = 3.67), although this did not reach statistical significance at the 0.05 level (*p* = 0.066), suggesting a possible trend. No significant associations were observed for age (*β* = −0.003, *p* = 0.973), metastasis status (*β* = 0.33, *p* = 0.904), or early stage disease (*β* = 2.09, *p* = 0.333), indicating that these clinical covariates did not contribute meaningfully to the observed differences (Table 2). A similar analysis employing a genetically dominant model (CC vs [CT + TT]) confirmed SLC22A4 genotype as the only relevant determinant of the outcome, although at a lower level of stringency (*p* = 0.0149, 95% CI: 1.06-9.76). (Supplementary Table 1).

**Table 2.** Multivariable regression analysis.

Variable	Beta	SE	*t*-value	*p*-value	CI 95%
C/T (vs CC)	3.67	1.99	1.84	0.066	−0.24; 7.57
T/T (vs CC)	11.10	3.16	3.51	**0.00045**	4.90; 17.30
Age	-0.003	0.074	-0.03	0.973	−0.15; 0.14
Metastasis (yes vs no)	0.33	2.70	0.12	0.904	−4.96; 5.61
Early stage (vs advanced)	2.09	2.16	0.97	0.333	−2.15; 6.33

Abbreviations SE: standard error; CI: confidence interval; *p* < 0.05 indicates statistical significance.

Bold values denote *p* < 0.05.

Thus, overall, the results support an effect of SLC22A4 variants on bacterial communities in tumor and healthy mucosa.

### SLC22A4 variants modulate innate immunity and *F. nucleatum* survival in colorectal cancer stem cells

**Figure 3.**
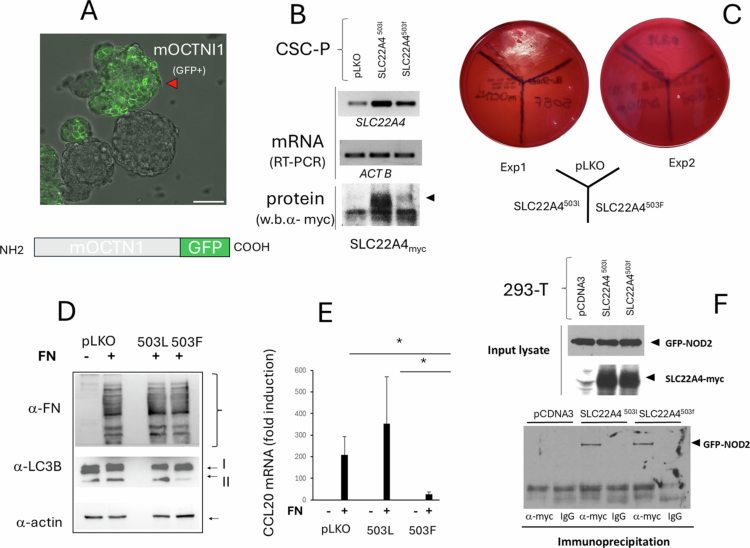
Effect of SLC22A4 variants on innate immunity and *F. nucleatum* clearance in colorectal cancer stem-like cells. (A). Lentiviral expression of murine, GFP-tagged SLC22A4 in Spheroidal cultures of colorectal cancer stem cells (CSC-P). The confocal photomicrograph confirms the expression and correct membrane localization of the recombinant mSLC22A4-GFP fusion protein in transduced cells. A schematic of the recombinant carrier is depicted in the lower panel. (B). RT-PCR (upper and middle panel) and immunoblotting (lower panel) analysis revealing endogenous expression of SLC22A4 and successful overexpression of the 503 L and 503F variants in CSC-*P* cells. CSC cDNA was amplified with primer sets specific for human SLC22A4 (SLC22A4) or Actin B, and the products were visualized by UV transillumination. Recombinant, myc-tagged SLC22A4 proteins were highlighted by anti-myc immunoblotting. Relevant bands are indicated by the arrows. (C). Gentamicin protection assay showing detectable *F. nucleatum* colonies only in 503F-bearing cells. Lysates of infected cells were plated on blood agar dishes and incubated in anoxic bags for 72-96 hours. A biological duplicate test with similar results is depicted. (D). The same protein homogenates as in C were subjected to western blotting to detect fusobacterial proteins (upper panel), LC3B I/II, and actin as a protein loading control. Decreased LC3B II signal in 503F cells is consistent with impaired Fn-induced autophagy. Representative of two independent experiments. (E). qPCR quantification of CCL20 mRNA in pLKO, 503 L, and 503F cells infected with *F. nucleatum.* mRNA abundance was assessed relative to actin by the 2^−^^ΔCt^ calculation method. Numbers are fold induction compared to the corresponding uninfected control. *N* = 5–8 independent experiments. Pairwise statistics by Mann–Whitney test. * denotes *p* < 0.02. n.s. = non-significant. (F). Co-immunoprecipitation of SLC22A4 variants with NOD2 in 293 T cells. Upper panel: western blot analysis of protein homogenates shows successful expression of myc-tagged SLC22A4 503 L and 503F variants (as indicated) together with a GFP-NOD2 fusion construct (all three populations). Relevant bands recognized by anti-GFP and anti-myc antisera are indicated by arrows. Lower panel: anti-GFP immunoblotting revealing the presence of GFP-NOD2 in anti-myc immunoprecipitates from SLC22A4-expressing cells. The absence of signal in mock immunoprecipitations (IgG) and in anti-myc immunoprecipitates from vector-transfected cells (pCDNA3, no SLC22A4) confirms the specificity of the NOD2-SLC22A4 interaction.

To gain mechanistic insights into the above SLC22A4-microbe-CRC connection, we used primary patient-derived colorectal cancer stem cells (P-CSCs) grown in vitro as undifferentiated spheroids.^27^ These cells are homozygous for the major 1672c allele and express low but detectable amounts of the transporter mRNA (Figure 3B, upper panel). Additionally, lentiviral transduction of a construct encoding mouse SLC22A4, C-terminally fused with GFP (mSLC22A4-GFP+, Figure 3A, lower scheme) confirmed the successful gene transfer and correct membrane localization of the recombinant carrier in these cells, as verified by fluorescence microscopy (Figure 3A, upper panel). In order to investigate the functional impact of the SLC22A4 503F variant in the epithelial tumor compartment, the human mutant allele (1672t), or the correspondent 1672c cDNA were transduced in CSC-P cells; a third control line was also created by transducing P-CSC cells with an empty lentiviral vector (pLKO.1). Protein immunoblotting confirmed the expression of the recombinant, myc-tagged carriers, with the 503F variant displaying lower expression levels compared to the 503 L isoform. The three lines were infected with 200 MOI of live *Fn* for 6 hours, followed by the addition of the non-cell-permeant antibiotic Gentamicin for a further 18 hours to kill extracellular bacteria and assess the survival of invading microbes within cells (Gentamicin Protection Assay). After cell lysis in low-detergent buffer, colony-forming bacteria could be recovered, albeit with a low yield, only from the variant-bearing cells, but not from the control line, nor from the line overexpressing the wild-type transporter (Figure 3C). Immunoblot analysis of total protein lysates from the three lines revealed similar signals for fusobacterial proteins (upper panel), confirming equal exposure to bacterial infection. Conversely, the lipidated form of Microtubule Associated Protein 1 Light Chain 3 (LC3B II), a marker of autophagy, was markedly reduced in the 503F line compared with the other two lines (Figure 3D), indicating reduced bacterial-killing capacity in this cell population.^28^ Likewise, upregulation of CCL20, a CRC-associated chemokine involved in leukocyte recruitment and direct anti-bacterial innate immunity,^29^ was also blunted in 503F-bearing cells compared to mock-transduced controls, while the opposite trend to increased expression was noted in the 503L-transduced cell population (Figure 3E). Finally, consistent with the role of SLC22A4 in intracellular pathogen sensing and innate immunity, both variants of the carrier formed complexes with the NOD-family receptor NOD2, a pattern recognition receptor previously shown to facilitate bacterial clearance by colonic epithelial cells,^30^ upon overexpression in 293 T cells. As a whole, these observations confirm that SLC22A4 variants affect the biological outcome of *F. nucleatum* interactions with colorectal cancer cells by attenuating innate immunity and promoting microbe intracellular survival.

## Discussion

The main findings presented here are that: (1) the abundance ratio between *F. nucleatum* and *E. coli*, instead of *F. nucleatum* abundance per se, is increased in CRC tumor tissue compared to the surrounding, seemingly normal mucosa; (2) the same calculated metric correlates with the CRC-associated SLC22A4 L503F variant, growing progressively through the C/C, C/T, and homozygous T/T patient groups; and (3) overexpression of SLC22A4-503F in stem-like colorectal cancer cells blunts the innate immune response to *F. nucleatum in vitro* and facilitates the survival of intracellular bacteria.

The presence and abundance of *F. nucleatum* in tumor and normal tissue are comparable in our cohort of CRC patients, although the number of cases in which *F. nucleatum* is more abundant in the tumor than in the corresponding normal mucosa slightly exceeds those showing the opposite (Figure 1A). This finding, in disagreement with many previous studies, may reflect the geographic and sociocultural peculiarities of the patient populations, a homogeneous cohort of individuals of the Muslim religion from North Africa. Notably, however, these data are not in conflict with an increased risk of CRC in *F. nucleatum* -colonized intestines, as data on *F. nucleatum* presence in the mucosa of a matched population of healthy individuals are not available to us. The finding of *F. nucleatum* in seemingly normal colon mucosa is by no means surprising, since, despite being frequently detected in higher quantities in tumorous tissues, *F. nucleatum* has also been reported in healthy tissues.^2^ Interestingly, its presence reportedly varies depending on the patient's inflammatory state or the particular composition of their intestinal microbiota.^31^ Unlike the absolute abundance of *F. nucleatum*, its representation relative to *E. coli* discriminates between cancerous and normal tissues in our cohort. This finding could have several explanations. From a statistical perspective, normalizing the *F. nucleatum* load for a highly represented fecal bacterial species may help mitigate confounding factors, such as fecal contamination, thereby improving data consistency. More attractively, the *F. nucleatum*/*E. coli* imbalance may better capture, compared to *F. nucleatum* load alone, the *F. nucleatum-associated* bacterial dysbiosis as a more distinctive hallmark of malignant tissue. Thus, the *F. nucleatum*/*E. coli* ratio warrants further validation in a larger population study; similarly, its biological significance must be better investigated mechanistically, since *E. coli* *per se* can act as a pathobiont and promote colon carcinogenesis.^31^ ^,^ ^32^

Our data also highlight a novel association between SLC22A4 and tumor-associated microbiota in CRC, suggesting an essential role for this transporter in regulating host-microbiota interactions inside the tumor microenvironment. Previous studies have shown that SLC22A4 variants may play a part in controlling the degree of chronic inflammation linked to advanced dysplasia and CRC in young people and UC patients, possibly via an enhanced mucosal inflammatory response that involves, in the context of the T/T genotype, NF-κB and the neutrophil chemokine IL-8.^21^ The finding of a steady increase in the *F. nucleatum*/*E. coli* ratio with the number of mutated T alleles (Figure 2B) is consistent with the hypothesis that changes in this transporter's function may alter the microbiota's equilibrium, promoting the growth of pathobionts like *F. nucleatum* at the expense of other, less harmful bacteria. This microbiological imbalance may ultimately foster colorectal cancer development. This view is further supported by our original observation that SLC22A4 physically interacts, at least under overexpression in 293 T cells, with the NOD-like microbial sensor NOD2, and by data showing an attenuated innate immune response to *F. nucleatum* in colorectal stem-like cancer cells engineered to express the tumor-associated variant 503F (Figure 3C). Of note, impaired intracellular bacterial killing and reduced levels of the lipidated form of LC3B (II) are consistent with impaired bacteria-induced autophagy in mutant cells, a defect shared by other genetic variants associated with chronic inflammatory bowel disease.^32^ Thus, by attenuating antibacterial responses, the SLC22A4 variant may foster the establishment of the symbiotic cancer-*F. nucleatum* interaction, eventually leading to CRC malignant progression.^33^

While the molecular mechanisms by which OCTN1SLC22A4 modulates the inflammatory response and bacterial clearance capacity of isolated epithelial cells *in vitro* remain elusive, solute carriers structurally related to SLC22A4 have been convincingly linked to innate immune signaling through NOD-like and Toll-like receptors.^34^ ^,^ ^35^ Accordingly, SLC22A4 forms a stable complex with NOD2 in 293 T cells (Figure 3 E), although this interaction does not appear modified in the 503F variant, at least at supranormal levels of expression. This evidence, together with observations implicating OCTNs in the cross-barrier transport of dietary antioxidants and of immuno-regulatory and “quorum sensing” factors released from intestinal flora,^36^ ^,^ ^37^ supports the hypothesis that genetic variants of these transporters shape the communication of host epithelial and/or inflammatory cells with microbes at the mucosal interface. Along this line of speculation, further molecular studies may validate SLC22A4 as a potential actionable target to interfere with *F. nucleatum's* action on malignant cells.

While novel and provocative, our study has several limitations. These are mainly related to the retrospective design, the preliminary nature of the observations, the geographically limited target population (entirely from North Africa), and the limited correlation between microbiological and clinico-pathological data. Additionally, the lack of *F. nucleatum* enrichment in our collection of tumors compared to the respective normal mucosa is an unexpected finding that remains to be fully explained. As for in vitro studies, while patient-derived epithelial CRC cells represent an excellent cellular model for tumor parenchyma, they cannot capture the complexity and heterogeneity of the tumor microenvironment and the contributions of stromal and immunoinflammatory cells. Finally, a detailed mechanism for how SLC22A4 and, especially, its human 503F variant affect *F. nucleatum* signaling and survival in cancer cells is still missing.

Notwithstanding the above limitations, our study has the merit of highlighting the importance of the host's genetic profile in microbial interactions that favor tumor development. Our approach is therefore perfectly aligned with the emerging field of molecular pathological epidemiology as a transdisciplinary method aimed at unraveling the intricate etiologic interactions among genetic variants, environmental exposures, and tumor molecular (and microbiological) profiles, that led to the “unique disease” of every single patient.^38^ Of note, genetic traits could be regarded as exposure variables that are intrinsically antecedent to disease development, thereby partially mitigating the limitations of a case-series-based retrospective experimental design. Mechanistically, our observations suggest a role for the intestinal transporter SLC22A4 in bacterial-host communication, with the genetic variant and its transported substrates affecting microbial ecology and promoting the establishment of the cancer-pathogen symbiotic relationship. Further research is warranted on the molecular underpinnings of the above interactions and their clinical relevance in larger cohorts of CRC patients. More specifically, the prospective cohort incident-tumor biobank method (PCIBM) has recently emerged as a new research design concept that couples the power of a prospective cohort study with an incident-tumor biobank to better capture the etiologic links between long-term exposure, individual susceptibility, and molecularly defined cancer subtypes.^39^ In the future, exploiting prospective cohort incident-tumor banks to investigate the SLC22A4/*F.nucleatum* axis, or similar combinations of immune germline variants with specific intratumor microbial communities, may greatly help clarify, from a personalized medicine perspective, the role of the intestinal microbiota in colorectal cancer genesis, evolution, and clinical outcomes.

## Supplementary Material

Supplementary Material Supplementary Information revised.docx

## Data Availability

This submission includes Supplementary Information. The data that support the findings of this study are openly available in FigShare, DOI: 10.6084/m9.figshare.31420841.
