# Supplementary material for: Genetic variants of the transporter SLC22A4 affect the abundance and survival of Fusobacterium nucleatum in colorectal cancer
Source: Gut Microbes. 2026 Jun 5;18(1):2681818. doi: 10.1080/19490976.2026.2681818 (PMC13245087; doi:10.1080/19490976.2026.2681818)
Supplement: Supplementary Material — Supplementary Information revised.docx [file KGMI_A_2681818_SM7320.docx]

**Supplementary Information.**


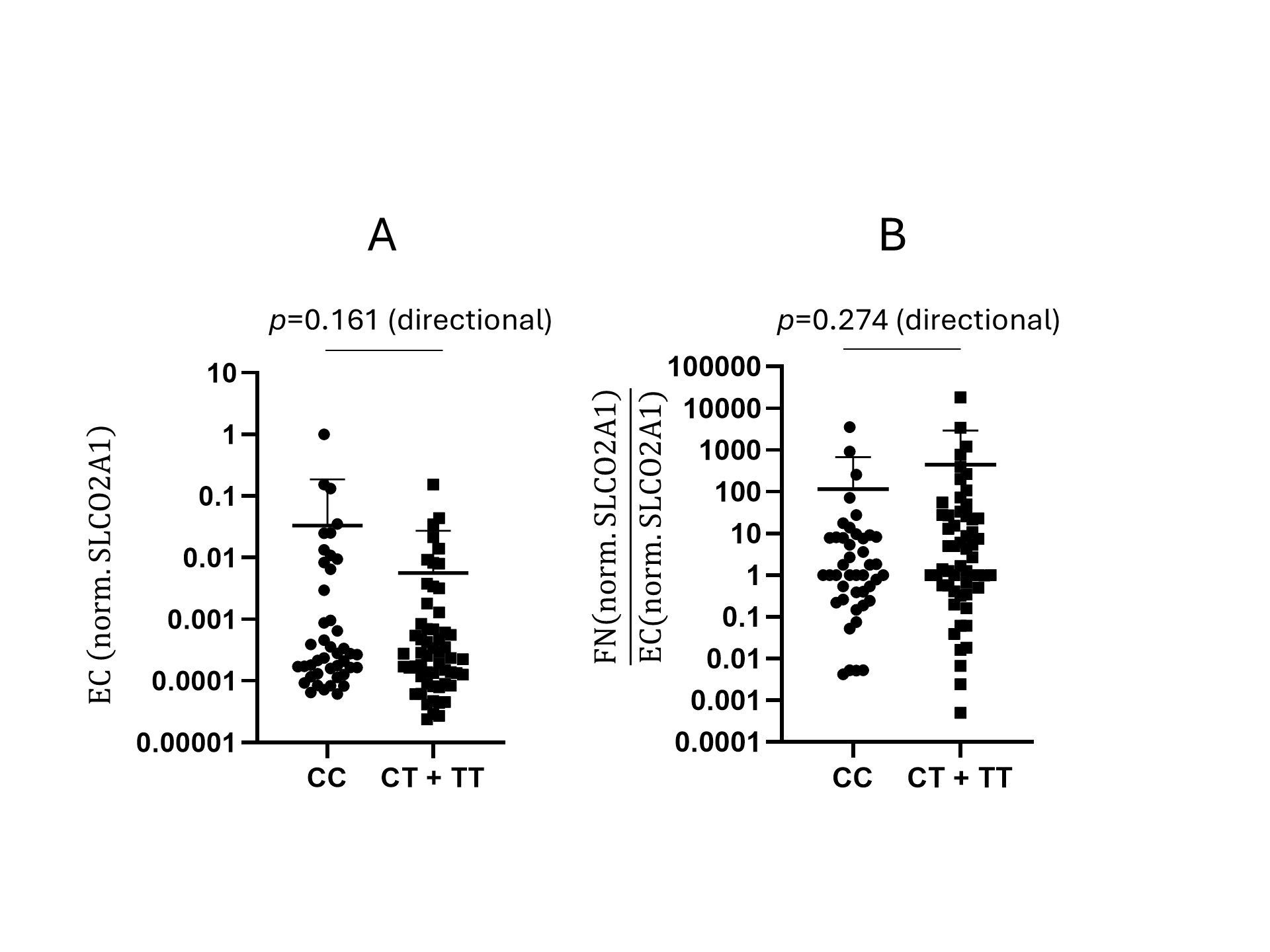


**Supplementary Figure 1: The IBD-associated SLC22A4 variant L503F shapes intratumor microbiota. A**. normalized *E. Coli* abundance in CRC patients grouped according to OCTN1 genotype under a dominant (0 *versus* one or two variant alleles) model. Each dot represents a tumor sample. CC (n=43); CT+TT (n=56). **B.** Distribution of the normalized *F. nucleatum / E. coli* abundance ratio across the same genotype groups as in A. A tendency towards decreased E. coli abundance and an increased F. nucleatum/E. coli ratio suggests an effect of the IBD-associated variant of the transporter on intratumor microbiota. Statistics by one-tailed Mann-Whitney test.

**Supplementary Table 1.** **Multivariable regression analysis. Recessive model.**

| **Variable** | **Beta** | **SE** | **t value** | **p value** | **CI 95%** |
| --- | --- | --- | --- | --- | --- |
| **Genotype TT/CT (vs CC)** | 5.41 | 2.22 | 2.44 | **0.0149** | 1.06 - 9.76 |
| Age | 0.04 | 0.09 | 0.44 | 0.659 | -0.13 - 0.21 |
| Metastasis (Yes vs No) | -0.50 | 3.17 | -0.16 | 0.875 | -6.72 - 5.72 |
| Early stage ( vs advanced) | 1.96 | 2.54 | 0.77 | 0.440 | -3.02 - 6.94 |

Abbreviations SE: standard error; CI: confidence interval; *p* < 0.05 indicates statistical significance.
